# Advances in hemodialysis therapy

**DOI:** 10.12703/r/12-12

**Published:** 2023-05-16

**Authors:** Bijin Thajudeen, Dany Issa, Prabir Roy-Chaudhury

**Affiliations:** 1 Division of Nephrology, Banner University of Arizona, 1501 N Campbell Ave, Tucson, AZ 85724, USA; 2 WG (Bill) Hefner VA Medical Center, 1601 Brenner Ave, Salisbury, NC 28144, USA; 2,3 UNC Kidney Center, University of North Carolina at Chapel Hill, 101 Manning Dr, Chapel Hill, NC 27514, USA

**Keywords:** End-stage renal disease, Hemodiafiltration, hemodialysis, renal replacement therapy

## Abstract

End-stage renal disease (ESRD) continues to be a disease process with a high rate of hospitalization and mortality. There has been little innovation in nephrology over the last few decades compared to revolutionary high-tech advancements in other areas like oncology and cardiovascular medicine. Kidney transplantation, the only available alternative to renal replacement therapy, is limited in its availability. It is essential to have advances in this field to improve the efficiency of currently available treatments and devise new therapies. The current description of renal replacement therapy is inappropriate as it only replaces the filtration function of the failed kidney without addressing its other vital metabolic, endocrinologic, and immunologic roles and portability. Hence, it is critical to have newer therapies focusing on total replacement and portability, not just clearance. This review will address the developments in hemodialysis therapy. Advances in hemodialysis therapy include hemodiafiltration, portable machines, wearable artificial kidneys, and bioartificial kidneys. Although promising, newer technologies in this direction are still far from clinical application. Several organizations and enterprises including the Kidney Health Initiative and Kidney X: The Kidney Innovation Accelerator, as well as The Advancing American Kidney Health Initiative, are working in tandem to develop new therapies that could customize the treatment of ESRD.

Since its initial use, hemodialysis has been continually refined to provide better outcomes for patients. However, although it was historically referred to as “renal replacement therapy”, this description may be overly broad, as the primary functions of dialysis are to remove uremic toxins, maintain electrolyte balance, and restore fluid balance without addressing its other vital metabolic, endocrine, and immune functions. Additionally, limitations of currently available hemodialysis membranes include the tendency to occlude over time because of protein deposition and thrombus formation and the lack of portability of both the device and the procedure^1^. Hence, it is vital to have newer therapies with a goal of total replacement and not just clearance and with a focus not just on improved survival but also on patient-centered needs such as enhanced portability, the flexibility of therapy, reduced interventions, improved quality of life, and a reduction in the economic impact of dialysis on the family and society as a whole^2^. The following paragraphs will summarize some of the recent advances in the field.

## a) Hemodiafiltration

Hemodiafiltration (HDF) functions on the principle of combining hemodialysis (movement of molecules across a membrane by diffusion) and hemofiltration (movement of molecules across a membrane along with movement of solvent) with the ability to remove both small and middle-size solutes^3^. Online HDF is a method of combining HDF with the online generation of sterile and nonpyrogenic solutions for fluid substitution^4^. HDF has been postulated to minimize intradialytic hypotension and reduce dialysis-related pathologies such as amyloidosis, accelerated atherosclerosis, and erythropoietin hypo-responsiveness, the last of which is likely related to better clearance of middle-sized uremic toxins, including erythropoietic inhibitor substances and the use of higher-quality water as well as dialysate, which reduces inflammation^5^. It has also been linked to improved nutritional status, reduced inflammation, and better preservation of residual renal function^3^. Despite these advantages, this technology has not gained acceptability in the United States, because of increased cost and regulatory issues stemming from the safety concerns regarding the online production of the large volumes of sterile, nonpyrogenic substitution fluid, which is infused directly into the bloodstream^6^. Additionally, it needs specially trained staff and good vascular access to accommodate high blood flow requirements^4^. In addition, clinical trials using HDF have shown mixed results. Although retrospective studies showed improved patient survival, prospective randomized controlled trials have not demonstrated overall survival advantage or impact on complications like neuropathy^7,8^.

## b) Portable hemodialysis machines

Over the years, several attempts have been made to reduce the size of the dialysis machine and to make it more portable. The earliest mobile devices used a batch dialysate system, and the spent dialysate was regenerated by a charcoal module with rechargeable batteries^9,10^. These devices were abandoned because of their large size and weight. More recently, a number of portable and compact machines have been developed by various manufacturers. These devices strive to improve patient autonomy by making it even easier for patients to perform dialysis at home (compared with older technologies), resulting in both freedom and flexibility. Moreover, these devices provide patients with the ability to make more personalized dietary choices and to leverage dialysis so they can live a more normal life by adjusting the treatment schedule that fits their lifestyle, instead of living their lives around dialysis, as so often occurs in the in-center setting where the schedule is set by the facility providing dialysis care rather than the patient.

## c) Wearable artificial kidney: Hemodialysis

In the 1970s, Kolff and colleagues were responsible for the development of the first portable and wearable dialysis system^11^. Indeed, to quote Dr. Kolff, “if the treatment of chronic uremia cannot rehabilitate people, then that treatment is inadequate”. Incorporation of sorbent technology into a wearable hemodialysis system proposed by Blumenkrantz and colleagues in the late 1970s paved the way for further research in this field^12^. Although Murisasco and colleagues developed a wearable hemofiltration device utilizing a sorbent-containing mini-cartridge, the device had a number of difficult-to-navigate limitations, including the requirement for excessive replacement fluid and the need for transcutaneous arterio-venous shunts, which are associated with a high risk of infections^12^. These initial technological innovations have led to the development of the wearable artificial kidney (WAK), which extends previous efforts through the incorporation of modern-day miniaturization technology, biocompatible dialyzer materials, battery design, and safety features^13^.

WAK is a device that combines a miniaturized dual-channel battery-operated pulsatile pump for driving blood and dialysate together with dialysate regenerative technology^13^. Ever since its initial conceptualization and design, Gura and colleagues developed different prototypes of WAK, and the most recent is WAK 3^14^. Short-duration pilot studies using prototype 1 have demonstrated safety and efficacy for both solute clearance and fluid removal^12,14^. A version 2.0 was developed with the intention of utilization by patients for up to 24 hours per day, serving as a wearable form of extended-hours hemodialysis compared with conventional thrice-weekly hemodialysis^12^. A clinical trial involving 10 patients was conducted for 24 hours. Unfortunately, owing to device-related technical problems, the study was abandoned. Technical issues noted included kinking of the tube, fluctuation in blood as well as dialysate flow rates, clotting, hemolysis, suboptimal dialysis due to ammonia saturation of the sorbent column, gas bubbles in the circuit, and problems with the battery. Moreover, five subjects were noted to have premature ventricular contractions^12^.

## d) Wearable artificial kidney: Peritoneal dialysis

Ronco and Fecondini introduced the concept of a WAK device for peritoneal dialysis (PD)^15^. This system consisted of a double-lumen peritoneal catheter, lines for dialysate inflow and outflow, a miniaturized rotary pump, and a circuit for dialysate regeneration. The system also has four sorbent cartridges in parallel, a filter for deaeration and microbiological safety, and a personal digital assistant as a remote control^15^. The Vicenza WAK is a portable PD system that uses a standard fresh glucose-based dialysate. The spent dialysate fluid is pumped through a sorbent filter and a degassing chamber before being returned to the patient. Subsequently, this fluid will be drained out, and a new bag of 7.5% icodextrin will be added to aid solute clearance and volume control. The need for additional fluid, however, negated the advantages of this system^15^.

Lee and Roberts have developed another model of WAK for PD, currently being commercialized as AWAK (automated wearable artificial kidney)^16^. This device can recycle peritoneal dialysate through a system that comprises urease enzymes that convert urea to ammonia, sorbents such as zirconium phosphate, which adsorb ammonia, potassium, calcium, and magnesium, as well as other cations, and zirconium carbonate, which absorbs hydrogen ions, phosphate, fluoride, and heavy metals^13^. A similar device using sorbent technology is the WEAKID, which is currently under development^16^.

## e) Sorbent devices in dialysis therapy

Sorbent devices work by direct adsorption and/or retention of molecules. They can remove endogenous and exogenous materials such as middle uremic toxins, protein-bound uremic toxins, hydrophobic or protein-bound exogenous substances, cytokines, complements, free hemoglobin, and residual drugs. Some of the sorbents take up molecules until they are saturated, whereas others function primarily by exchanging one molecule for another. A significant limitation of such devices was biocompatibility^1^. However, over the years, there has been tremendous progress in the development of biocompatible sorbents. Current sorbent particles have the ability to remove the impurities from the dialysate, aiding in the regeneration and reuse of dialysate fluid. Prospective and observational studies involving close to 100 patients have investigated the effects of adding sorbent cartridges to conventional hemodialysis and found that the combination resulted in less pruritis, decreased parathyroid hormone and calcium-phosphate product, and an improved quality of life in addition to survival rate^17^.

## f) Bio-artificial kidney

Cell-based therapeutics work on the principle that cells that have been damaged or are dysfunctional in various disease states can be replaced^18^. This technology is the basis for the development of a bio-artificial kidney. Like the human kidney, the bio-artificial organ produces an ultrafiltrate that undergoes special processing resulting in the reabsorption of the majority of water and electrolytes in addition to the excretion of concentrated waste. The addition of metabolic activity, antioxidant activity, production of vitamin D_3_ as well as erythropoietin, immunoregulation, and cytokine homeostasis allows the replacement of critical physiologic functions of the kidney^19^. The combination of glomerular membrane function along with tubular, metabolic, and endocrine function could change the current natural history of the disease process of end-stage kidney disease (ESKD). The replacement of these critical moieties may reduce the inflammation associated with ESKD but also may mitigate the accelerated atherogenesis in patients with ESKD and may minimize the progression of renal osteodystrophy^20^. Like the transplanted kidney, these bio-artificial kidney models can be implanted into the human body but without the need for immunosuppression.

An ideal bio-artificial kidney, therefore, should have the following traits: be small and light-weight, have the ability to regulate acid–base as well as electrolyte balance, address metabolic and endocrine functions, and (most importantly) maintain appropriate safety mechanisms (especially for volume). Some examples of bio-artificial kidneys are the renal assist device, the bio-artificial renal epithelial cell system (BRECS), the human nephron filter, and the implantable kidney (the Kidney Project).

### 1) The renal assist device

The renal assist device is based on the ability to isolate and grow adult tubular cells in culture. These living cells can replace the function of tubules when added to a hemodialysis circuit^21^. They are grown along the inner surface of the fibers of the hemofiltration cartridge, which is then positioned in series with a conventional hemofilter. The hemofiltration cartridge uses silicone nanopore membranes, which can function like the glomerulus of native kidney^22^. These silicone membranes can perform filtration at the rate of 30 mL/min without assistance from an internal pump. The tubular cells seeding the cartridge behave much like native kidney tubules by reabsorbing water as well as solutes, excreting waste, and performing intrinsic metabolic, endocrine, and immunomodulatory functions^19^. Clinical trials with the renal assist devices conducted in animals and critically ill patients have produced promising cardiovascular and survival benefits^19,23^.

### 2) The bio-artificial renal epithelial cell system

The BRECS comprises a dense population of adult human renal epithelial cells grown on porous, niobium-coated carbon disks within a bioreactor housing. After the cells reach an optimum density, the BRECS can be cryopreserved, transported, and stored at a clinical site for on-demand use in hemodialysis or PD circuits. They function similarly to the renal assist device. The innovative combination of an implanted hemofilter and an implantable BRECS would provide continuous small-solute clearance along with metabolic functions of the proximal tubule^24^.

### 3) Human nephron filter

The human nephron filter utilizes nanotechnology. The membrane cartridge is part of a wearable system that includes a keypad and display, a high-capacity battery, and a waste bag. It has two types of membranes: the glomerular and the tubular membrane. The ultrafiltrate produced by the glomerular membrane passes over the tubular membrane, recovering most of the desirable solutes and rejecting unwanted ones. This model ensures results in a glomerular filtration rate equivalent to 30 mL/min. The system can also reduce levels of middle molecules, including β2-microglobulin, compared with other dialytic approaches^25^.

### 4) The Kidney Project

The Kidney Project aims to use silicon nanomembranes as the filtration system and proximal tubular cells coated onto these silicon nanomembranes as the resorptive mechanism. Recent experiments have documented that this implantable kidney can, in fact, be connected to the femoral artery and vein in a pig and produce a filtrate that provides renal clearance^22,26–29^.

## g) Creating an innovation substrate to advance patient-centered renal replacement therapy

Innovation, of course, does not occur in isolation; rather it needs an innovation substrate that goes all the way from biology to experimental and clinical studies to investment to regulatory and reimbursement pathways. In this context, we are currently in an advantageous position for innovative kidney replacement therapy (KRT) as a result of a confluence of three linked initiatives that aim to achieve this goal. These initiatives are the following:

(a) the Kidney Health Initiative (KHI), which is a public/private partnership between the American Society of Nephrology (ASN) and the US Food and Drug Administration with a mandate to facilitate the passage of drugs, devices, and biologics into the kidney disease space^30,31^. The KHI aims to achieve this by carrying out projects, one of which focuses on the creation of an innovative patient-centered KRT roadmap that goes all the way from advanced dialysis to the creation of portable, wearable, implantable, and bioengineered kidneys^2^.
(b) the Kidney Innovation Accelerator (Kidney X), which is a public/private partnership between the ASN and the US Department of Health and Human Services, which is currently running prize competitions in the kidney disease area with a focus on an artificial kidney prize^32,33^.
(c) the Advancing American Kidney Health Initiative^34–36^, which is an executive order that is prioritizing home dialysis.

In summary, we are truly in a unique position of overt enthusiasm regarding innovation, investment, and excitement in this area and hope that the next decade will truly change the way we provide KRT. At the same time, we have to leverage the moment; otherwise, our patients will look at the multitude of new and effective therapies in other disease conditions and ask us “if not you, who? If not now when?”

## References

[1] Cruz D Bellomo R Kellum JA : The future of extracorporeal support. *Crit Care Med.* 2008; 36(4 Suppl): S243–52. 10.1097/CCM.0b013e318168e4f6 18382201

[2] Bonventre JV Hurst FP West M : A Technology Roadmap for Innovative Approaches to Kidney Replacement Therapies: A Catalyst for Change. *Clin J Am Soc Nephrol.* 2019; 14(10): 1539–47. 10.2215/CJN.02570319 31562182 PMC6777588

[3] Canaud B Vienken J Ash S : Hemodiafiltration to Address Unmet Medical Needs ESKD Patients. *Clin J Am Soc Nephrol.* 2018; 13(9): 1435–43. 10.2215/CJN.12631117 29511057 PMC6140578

[4] Ledebo I : On-Line Hemodiafiltration: Technique and Therapy. *Adv Ren Replace Ther.* 1999; 6(2): 195–208. 10.1016/s1073-4449(99)70038-5 10230890

[5] Panichi V Scatena A Rosati A : High-volume online haemodiafiltration improves erythropoiesis-stimulating agent (ESA) resistance in comparison with low-flux bicarbonate dialysis: Results of the REDERT study. *Nephrol Dial Transplant.* 2015; 30(4): 682–9. 10.1093/ndt/gfu345 25385719

[6] Ward RA Vienken J Silverstein DM : Regulatory Considerations for Hemodiafiltration in the United States. *Clin J Am Soc Nephrol.* 2018; 13(9): 1444–9. 10.2215/CJN.12641117 29511058 PMC6140579

[7] Grooteman MPC van den Dorpel MA Bots ML : Effect of online hemodiafiltration on all-cause mortality and cardiovascular outcomes. *J Am Soc Nephrol.* 2012; 23(6): 1087–96. 10.1681/ASN.2011121140 22539829 PMC3358764

[8] Kang A Arnold R Gallagher M : Effect of Hemodiafiltration on the Progression of Neuropathy with Kidney Failure: A Randomized Controlled Trial. *Clin J Am Soc Nephrol.* 2021; 16(9): 1365–75. 10.2215/CJN.17151120 34233923 PMC8729572

[9] Gordon A Greenbaum MA Marantz LB : A sorbent based low volume recirculating dialysate system. *Trans Am Soc Artif Intern Organs.* 1969; 15: 347–52. 5791408

[10] Dharnidharka SG Kirkham R Kolff WJ : Toward a wearable artificial kidney using ultrafiltrate as dialysate. *Trans Am Soc Artif Intern Organs.* 1973; 19: 92–7. 10.1097/00002480-197301900-00018 4722782

[11] Kim JC Garzotto F Nalesso F : A wearable artificial kidney: Technical requirements and potential solutions. *Expert Rev Med Devices.* 2011; 8(5): 567–79. 10.1586/erd.11.33 22026622

[12] Gura V Rivara MB Bieber S : A wearable artificial kidney for patients with end-stage renal disease. *JCI Insight.* 2016; 1(8): e86397. 10.1172/jci.insight.86397 27398407 PMC4936831

[13] Davenport A : Portable and wearable dialysis devices for the treatment of patients with end-stage kidney failure: Wishful thinking or just over the horizon? *Pediatr Nephrol.* 2015; 30(12): 2053–60. 10.1007/s00467-014-2968-3 25330876 PMC4623087

[14] https://www.healio.com/news/nephrology/20210511/us-issues-patent-for-wearable-artificial-kidney

[15] Ronco C Fecondini L : The Vicenza wearable artificial kidney for peritoneal dialysis (ViWAK PD). *Blood Purif.* 2007; 25(4): 383–8. 10.1159/000107775 17785968

[16] Htay H Gow SK Jayaballa M : Preliminary safety study of the Automated Wearable Artificial Kidney (AWAK) in Peritoneal Dialysis patients. *Perit Dial Int.* 2022; 42(4): 394–402. 10.1177/08968608211019232 34105417

[17] Ankawi G Fan W Pomarè Montin D : A New Series of Sorbent Devices for Multiple Clinical Purposes: Current Evidence and Future Directions. *Blood Purif.* 2019; 47(1–3): 94–100. 10.1159/000493523 30253409

[18] Kim S Fissell WH Humes DH : Current strategies and challenges in engineering a bioartificial kidney. *Front Biosci (Elite Ed).* 2015; 7(2): 215–28. 10.2741/E729 25553375 PMC4383659

[19] van Gelder MK Mihaila SM Jansen J : From portable dialysis to a bioengineered kidney. *Expert Rev Med Devices.* 2018; 15(5): 323–36. 10.1080/17434440.2018.1462697 29633900

[20] Buffington DA Westover AJ Johnston KA : The bioartificial kidney. *Transl Res.* 2014; 163(4): 342–51. 10.1016/j.trsl.2013.10.006 24269374

[21] Humes HD MacKay SM Funke AJ : Tissue engineering of a bioartificial renal tubule assist device: *In vitro* transport and metabolic characteristics. *Kidney Int.* 1999; 55(6): 2502–14. 10.1046/j.1523-1755.1999.00486.x 10354300

[22] Kensinger C Karp S Kant R : First Implantation of Silicon Nanopore Membrane Hemofilters. *ASAIO J.* 2016; 62(4): 491–5. 10.1097/MAT.0000000000000367 26978710 PMC4983406

[23] Issa N Messer J Paganini EP : Renal assist device and treatment of sepsis-induced acute kidney injury in intensive care units. *Contrib Nephrol.* 2007; 156: 419–27. 10.1159/000102133 17464153

[24] Johnston KA Westover AJ Rojas-Pena A : Development of a wearable bioartificial kidney using the Bioartificial Renal Epithelial Cell System (BRECS). *J Tissue Eng Regen Med.* 2017; 11(11): 3048–55. 10.1002/term.2206 27860413

[25] Nissenson AR Ronco C Pergamit G : Continuously functioning artificial nephron system: The promise of nanotechnology. *Hemodial Int.* 2005; 9(3): 210–7. 10.1111/j.1492-7535.2005.01135.x 16191071

[26] Hojs N Fissell WH Roy S : Ambulatory Hemodialysis-Technology Landscape and Potential for Patient-Centered Treatment. *Clin J Am Soc Nephrol.* 2020; 15(1): 152–9. 10.2215/CJN.01970219 31727617 PMC6946084

[27] Fissell WH Roy S : Treating the kidneys - a new era in the United States (and beyond). *Nat Rev Nephrol.* 2019; 15(12): 727–8. 10.1038/s41581-019-0209-5 31541213

[28] Salani M Roy S Fissell WH 4th : Innovations in Wearable and Implantable Artificial Kidneys. *Am J Kidney Dis.* 2018; 72(5): 745–51. 10.1053/j.ajkd.2018.06.005 30146422

[29] Feinberg BJ Hsiao JC Park J : Silicon nanoporous membranes as a rigorous platform for validation of biomolecular transport models. *J Memb Sci.* 2017; 536: 44–51. 10.1016/j.memsci.2017.04.030 28936029 PMC5604868

[30] Linde PG Archdeacon P Breyer MD : Overcoming Barriers in Kidney Health-Forging a Platform for Innovation. *J Am Soc Nephrol.* 2016; 27(7): 1902–10. 10.1681/ASN.2015090976 27127187 PMC4926987

[31] Archdeacon P Shaffer RN Winkelmayer WC : Fostering innovation, advancing patient safety: The kidney health initiative. *Clin J Am Soc Nephrol.* 2013; 8(9): 1609–17. 10.2215/CJN.01140113 23744001 PMC3805066

[32] ASN Staff: KidneyX Summit Announces Winners of the Redesign Dialysis Phase 2 Prize. Kidney News, 2020. Reference Source

[33] HHS Names KidneyX Redesign Dialysis Phase I Winners. 2019. Reference Source

[34] Mehrotra R : Advancing American Kidney Health: An Introduction. *Clin J Am Soc Nephrol.* 2019; 14(12): 1788. 10.2215/CJN.11840919 31690694 PMC6895493

[35] Knight R : A Patient's Perspective on Advancing American Kidney Health Initiative. *Clin J Am Soc Nephrol.* 2019; 14(12): 1795–7. 10.2215/CJN.10560919 31694863 PMC6895484

[36] Bieber SD Gadegbeku CA : A Call to Action for the Kidney Community: Nephrologists' Perspective on Advancing American Kidney Health. *Clin J Am Soc Nephrol.* 2019; 14(12): 1799–801. 10.2215/CJN.10470919 31699812 PMC6895494

